# Prevalence and factors associated with os acromiale: a multicenter study

**DOI:** 10.1016/j.jseint.2025.05.015

**Published:** 2025-06-02

**Authors:** Naoya Kozono, Akihiro Nishii, Eiichi Ishitani, Yasuhiro Mizuki, Takehiro Kimura, Shunsaku Yamamoto, Naohide Takeuchi, Hidehiko Yuge, Kunio Iura, Akira Nabeshima, Yuta Sakemi, Eiji Tashiro, Erina Yamada, Kenji Takagishi, Yasuharu Nakashima

**Affiliations:** a Department of Orthopaedic Surgery, Graduate School of Medical Sciences, Kyushu University, Fukuoka, Japan; b Department of Orthopaedic Surgery, Kitakyushu Municipal Medical Center, Kitakyushu, Japan; c Department of Orthopaedic Surgery, Fukuoka Shion Hospital, Fukuoka, Japan; d Department of Orthopaedic Surgery, Sasebo Kyosai Hospital, Nagasaki, Japan; e Department of Orthopaedic Surgery, Moro-oka Orthopaedic Hospital, Fukuoka, Japan; f Department of Orthopaedic Surgery, Koga Hospital 21, Kurume, Japan; g Department of Orthopaedic Surgery, Harasanshin Hospital, Fukuoka, Japan; h Department of Orthopaedic Surgery, Mizoguchi Orthopaedic Hospital, Fukuoka, Japan; i Department of Orthopaedic Surgery, Fukuoka Orthopaedic Surgery, Fukuoka, Japan; j Department of Orthopaedic Surgery, Sada Hospital, Fukuoka, Japan

**Keywords:** Os acromiale, Prevalence, Rotator cuff, Shoulder, Magnetic resonance imaging, Injury

## Abstract

**Background:**

Os acromiale is an accessory bone resulting from the nonunion of the acromial ossification center, with a reported prevalence of approximately 2%. While os acromiale is often asymptomatic, when symptomatic, it has been reported to be strongly associated with rotator cuff injuries. This multicenter study aimed to determine the prevalence of and the factors associated with os acromiale in the Japanese population.

**Methods:**

We analyzed 6,842 shoulder magnetic resonance imaging scans for shoulder disabilities obtained at 10 facilities between April 2018 and March 2023. The average age was 63.7 years, with 3,483 male and 3,359 female shoulders. This study assessed the prevalence of os acromiale, its location (pre-acromion, meso-acromion, and meta-acromion), size (length, width, and thickness), acromion shape (square tip, intermediate, and cobra), and the association between os acromiale and rotator cuff injuries. Statistical analysis was performed using Fisher's exact test with a significance level set at *P* < .05.

**Results:**

A total of 76 cases of os acromiale (1.1%) were identified. There were 58 shoulders with pre-acromion, 18 with meso-acromion, and none with meta-acromion. The dimensions were as follows: pre-acromion (length 7.5 mm, width 10.5 mm, and thickness 5.3 mm), and meso-acromion (length 20.1 mm, width 20.8 mm, and thickness 8.5 mm). Acromial shapes included a square tip in 13 shoulders, intermediate tip in 38 shoulders, and cobra shape in 25 shoulders. The presence of os acromiale was significantly associated with rotator cuff injury (*P* < .001).

**Conclusion:**

The study supports previous findings that os acromiale is associated with rotator cuff injuries. In addition, the prevalence of os acromiale in the Japanese population was lower than that in non-Asian populations, and the size of os acromiale tended to be smaller than in European populations.

Os acromiale is an accessory bone resulting from the nonunion of the acromial ossification center, with a reported prevalence of approximately 2%.^5^^,^^10^^,^^11^ Os acromiale remains an underrecognized anatomical variant that can have significant clinical implications. The acromial apophysis develops from 4 distinct ossification centers: pre-acromion, meso-acromion, meta-acromion, and basi-acromion, with nonunion possible at the junction of any 2 centers (Fig. 1).^10^^,^^25^ The acromial ossification center forms by the age of 18 years and subsequently grows and fuses with the basi-acromion to form the definitive acromion between the ages of 23 and 25 years.^4^^,^^10^^,^^11^

**Figure 1:**
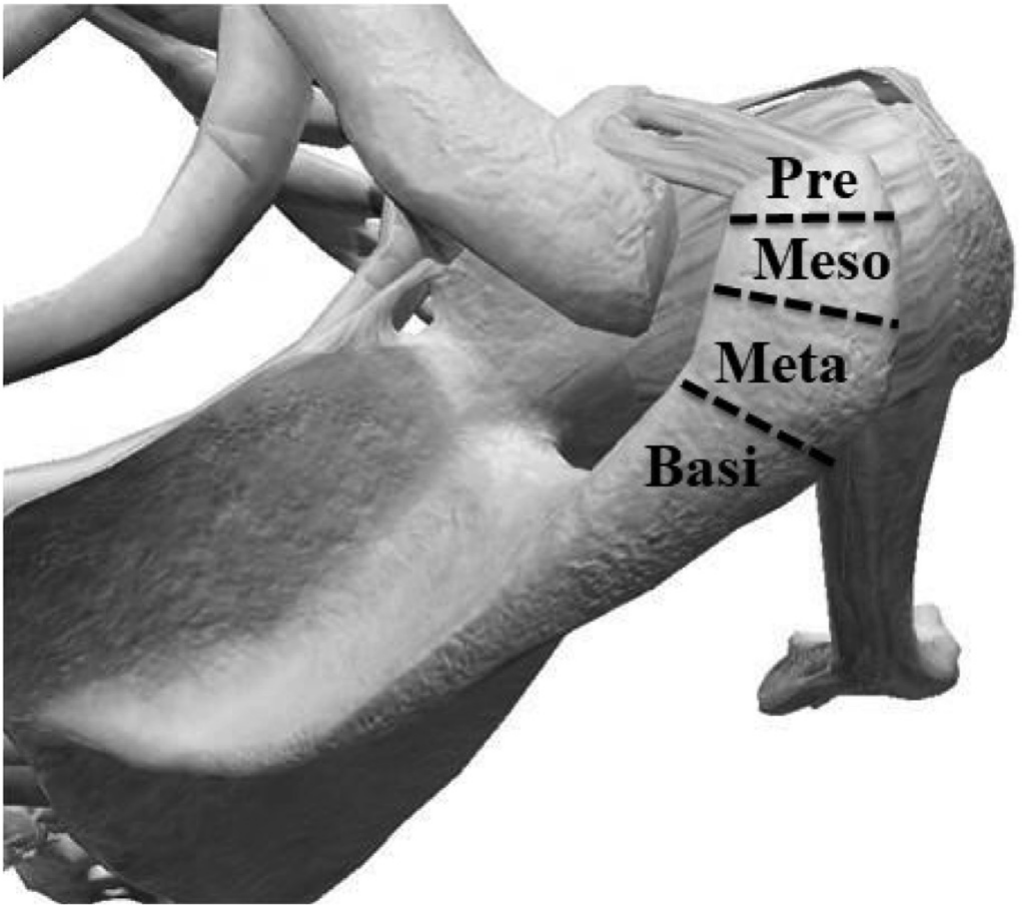
Three-dimensional model of the right shoulder illustrating the pre-acromion, meso-acromion, meta-acromion, and basi-acromion.

The acromion, located on the anterolateral aspect of the scapula, articulates medially with the lateral border of the clavicle. Beneath the acromion, the humeral head is separated from the rotator cuff (RC) muscles and the subacromial bursa. Generally, os acromiale alone is often asymptomatic, but its presence may be associated with an increased risk of RC injuries.^3^^,^^8^^,^^12^^,^^23^ However, the relationship between os acromiale and RC pathology remains controversial.^19^^,^^25^ The clinical significance of os acromiale lies in its potential association with RC-related pain, as the instability of unfused fragments may theoretically contribute to increased subacromial impingement. The diagnosis of os acromiale requires imaging studies, such as radiography, magnetic resonance imaging (MRI), or computed tomography (CT), of the shoulder joint.^3^^,^^15^ Several studies have used MRI as the gold standard for detecting the presence of os acromiale.^5^^,^^15^^,^^17^

Many orthopedic surgeons are interested in studying the os acromiale due to its critical role in shoulder motion, where the normal acromion provides attachment points for various muscles and ligaments. Several authors emphasize the importance of identifying os acromiale before surgery, as failure to recognize or address this condition may increase the risk of persistent pain or recurrence of RC pathology.^1^^,^^2^^,^^4^ However, in clinical practice, studies on reverse total shoulder arthroplasty suggest that preoperative acromial pathology, including os acromiale, acromial fragmentation, or severe thinning, does not significantly affect postoperative clinical outcomes.^20^^,^^22^ The unexpected finding of comparable outcomes despite preoperative acromial pathology might be explained by 2 factors. First, a recent biomechanical study demonstrated that scapulothoracic motion is more critical than glenohumeral motion following reverse total shoulder arthroplasty.^21^ Second, it is hypothesized that postoperative pain is not induced, as there is no RC to impinge against the acromion after reverse total shoulder arthroplasty.

In Japan and other Asian populations, os acromiale has been associated with unique morphological attributes, including a smaller size and distinct shapes compared to Western populations.^5^^,^^9^^,^^17^ The prevalence rates of os acromiale vary globally, ranging from 0.7% in Asian populations to as high as 18.2% in African cohorts.^4^^,^^9^ These variations highlight the role of ethnicity, environmental factors, and diagnostic methods. Population-specific investigations are justified, as they may elucidate morphological characteristics or associations that remain undetected in other ethnic groups. To the best of our knowledge, no previous study has assessed the prevalence of os acromiale in a Japanese population. The lack of data specific to Japan underscores the need for further research to understand the prevalence, anatomical variations, and clinical relevance of os acromiale in this population. Therefore, we aimed to determine the prevalence of and factors associated with os acromiale in the Japanese population.

## Materials and methods

This study was approved by the Institutional Review Boards (Approval Number: 23135-01) of Kyushu University Hospital and Medical Institutions, which served as the central ethics committee for all participating institutions. Given the retrospective nature of the study, informed consent was waived, and an opt-out consent model was implemented in accordance with ethical guidelines. Patients were provided with an opportunity to decline participation through publicly accessible notifications at each participating institution.

A total of 6,643 patients were recruited from 10 participating institutions between April 2018 and March 2023. All patients with shoulder symptoms (6,842 shoulders) underwent MRI scans. The inclusion criteria were (1) Japanese patients aged ≥20 years and (2) the requirement of MRI to investigate shoulder disorders. Patient demographic information, including sex, age, and associated shoulder diagnoses, was extracted from institutional databases.

**Figure 2:**
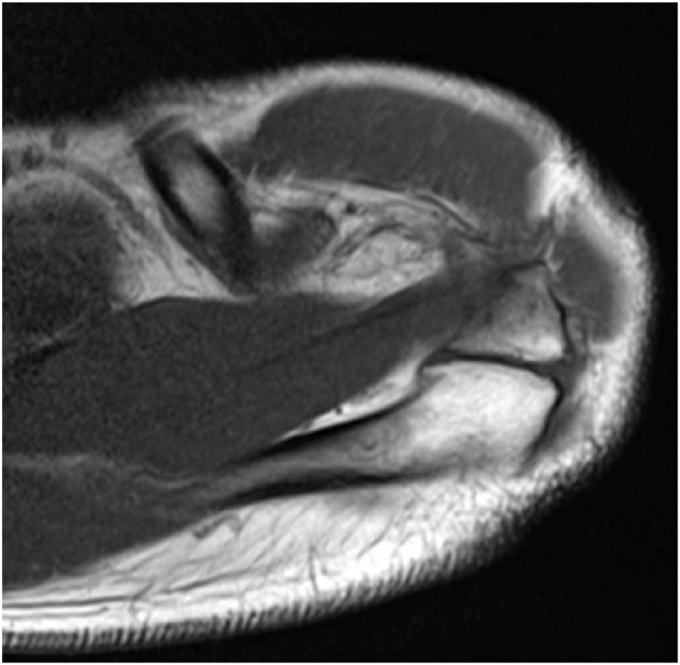
Os acromiale as seen on a T1-weighted magnetic resonance imaging axial view image.

MRI examinations were conducted using either a 1.5-T or 3-T system at the 10 participating institutions. All MRI scans were systematically reviewed and evaluated locally. The evaluations focused on identifying os acromiale and included several key parameters.^5^^,^^18^ (1) Location: Os acromiale was categorized into pre-acromion, meso-acromion, and meta-acromion (Fig. 1).^10^^,^^25^ Nonunion between the pre-acromion and meso-acromion was classified as pre-os acromiale, nonunion between the meso-acromion and meta-acromion was classified as meso-os acromiale, and nonunion between the meta-acromion and basi-acromion was classified as meta-os acromiale.^5^^,^^18^ (2) Size: Measurements included length, width, and thickness.^5^^,^^18^ (3) Acromion shape: Shapes were classified into square tip, intermediate, or cobra morphology, following Gumina et al.^6^ Shoulders with confirmed or borderline cases of os acromiale were reevaluated by 3 orthopedic surgeons (N.K., E.T., and E.Y.). In addition, MRI was used to evaluate the condition of the RC. A representative MRI image of the os acromiale is shown in Figure 2. The primary outcome of this study was the prevalence of os acromiale. The secondary outcomes included (1) morphological classification (location, size, and shape), (2) the association between os acromiale and RC injuries, and (3) laterality and sex-based distribution patterns.

### Statistical analysis

Case numbers and corresponding shoulder diagnoses are presented as counts and percentages, while demographic variables such as sex and age are shown as mean values with standard deviations. The correlation between the presence of os acromiale and RC injuries was analyzed using the Fisher exact test. Statistical significance was defined as *P* < .05. All analyses were performed using the JMP statistical software (version 17; SAS Institute, Cary, NC, USA).

## Results

Among the 6,643 patients (3,377 males and 3,266 females), the mean age was 63.7 ± 13.6 years. Os acromiale was identified in 76 of the 6,842 shoulders (3,927 right and 2,915 left), corresponding to a prevalence of 1.1%. Notably, among the 199 patients (106 males and 93 females) who underwent bilateral shoulder MRI, no cases of bilateral os acromiale were observed. The distribution of os acromiale cases was as follows: 58 cases (76.3%) were classified as pre-os acromiale, 18 cases (23.7%) as meso-os acromiale, and no cases (0%) as meta-os acromiale (Table I). The mean size of the pre-acromion fragment was 7.5 mm (length) × 10.5 mm (width) × 5.3 mm (thickness), while the mean size of the meso-acromion fragment was 20.1 mm (length) × 20.8 mm (width) × 8.5 mm (thickness) (Table II). Based on the classification by Gumina et al,^6^ square tip was observed in 13 cases (17.1%), intermediate in 38 cases (50.0%), and cobra in 25 cases (32.9%; Table II).

**Table I:** Frequency of os acromiale.

	Total	Male	Female
Number of patients	6,643	3,377	3,266
Mean age (year)	63.7 ± 13.6	62.1 ± 14.3	65.3 ± 12.7
Total examined shoulders	6,842	3,483	3,359
Right	3,927	1,953	1,974
Left	2,915	1,530	1,385
Os acromiale	76 (1.11%)	35 (1.00%)	41 (1.22%)
Right	46 (0.67%)	20 (0.57%)	26 (0.77%)
Left	30 (0.44%)	15 (0.43%)	15 (0.45%)
Classification of os acromiale			
Pre-os acromiale	58 (0.85%)	23 (0.66%)	35 (1.04%)
Meso-os acromiale	18 (0.26%)	12 (0.34%)	6 (0.18%)
Meta-os acromiale	0 (0.00%)	0 (0.00%)	0 (0.00%)

**Table II:** Mean size and Gumina classification of os acromiale.

	Length (mm)	Width (mm)	Thickness (mm)	Gumina classification
Pre-acromion (n = 58)	7.5 ± 3.5	10.5 ± 3.2	5.3 ± 1.5	Square tip: 5 Intermediate: 35 Cobra: 18
Meso-acromion (n = 18)	20.1 ± 6.3	20.8 ± 6.5	8.5 ± 2.6	Square tip: 8 Intermediate: 3 Cobra: 7

In total, 4,060 cases (59.3%) were diagnosed with RC injuries, while 2,782 cases (40.7%) were classified as non-RC injuries. Os acromiale was identified in 62 patients (81.6%) in the RC injury group and 14 patients (18.4%) in the non-RC injury group (Table III). Pre-os acromiale was observed in 48 patients with RC injury and 10 without RC injury, whereas meso-os acromiale was found in 14 patients with RC injury and 4 without RC injury. A significant difference was observed between patients with and without os acromiale when RC injuries were examined (*P* < .001).

**Table III:** Relationship between os acromiale and rotator cuff injuries.

	Rotator cuff injuries		
	Present	Absent	
Os acromiale			
Present	62 (81.6%)	14 (18.4%)	76 (100.0%)
Absent	3,998 (59.1%)	2,768 (40.9%)	6,766 (100.0%)
	4,060 (59.3%)	2,782 (40.7%)	6,842 (100.0%)

## Discussion

In this multicenter study, os acromiale was identified in 1.1% of the 6,842 shoulders (6,643 patients) examined. The prevalence reported here closely aligns with the rates documented previously in Asian populations,^9^^,^^17^ but it is significantly lower than the rates documented in Western and American populations.^4^^,^^5^^,^^24^ Gumina et al reported a prevalence of 4.9% in a cohort of 222 participants in Italy.^6^ The variability in prevalence across regions likely reflects differences in the ethnic composition of populations and the methodological approaches used in these studies.^5^^,^^6^^,^^9^^,^^17^ The highest prevalence of os acromiale has been reported in African-Americans and Africans, with rates ranging from 8.3% to 18.2%,^4^^,^^7^ and a higher incidence of bilateral cases has been noted in Black individuals.^7^^,^^24^

Historically, studies conducted between the 1990s and 2000s primarily relied on skeletal specimens and radiographs for diagnosis.^3^^,^^4^^,^^6^^,^^7^ However, since the 2010s, there has been a growing preference for advanced imaging techniques such as MRI, which offer improved diagnostic accuracy.^5^^,^^9^^,^^17^ In the present study, MRI was used for detection because it is widely recognized as the most reliable imaging modality for identifying os acromiale with high sensitivity and accuracy. Despite the use of this advanced imaging modality, the prevalence of os acromiale in the Japanese cohort remained lower than that reported in Western and American populations.^4^^,^^5^

As reported in a Thai study, the majority of os acromiale cases identified in the present study were classified as pre-os acromiale (76.3%).^17^ In contrast, several studies have indicated that the majority of os acromiale cases were predominantly classified as meso-os acromiale.^5^^,^^6^^,^^7^^,^^9^^,^^18^ Based on these data, it can be hypothesized that a genetics-based multifactorial pattern associated with ancestry plays a significant role in the development of os acromiale. In particular, substantial variation can be observed in growth trajectories and acromial development among individuals. In this study, the size of os acromiale in the Japanese population was found to be smaller compared to data reported from Western populations.^5^ Morphologically, the intermediate type (50.0%) was the most prevalent, consistent with findings from Western populations.^5^ Gumina et al^6^ proposed that the prevalence of os acromiale is positively correlated with the distance between the anterior aspect of the acromion and the acromioclavicular joint, suggesting a potential anatomical predisposition to its development. However, the present study did not support this hypothesis, as most cases of os acromiale were classified as the intermediate type based on the Gumina classification (50.0%). The smaller size of the os acromiale observed in this study is likely attributable to differences in overall body size. The Japanese population, classified as East Asians, has a lower average height and body weight compared to Caucasian populations. Recognizing these distinctions is important, as they may significantly influence clinical presentation and inform surgical treatment strategies.

Moreover, the presence of os acromiale was significantly associated with diseases involving RC injuries. These findings align with previous studies that have reported a relationship between os acromiale and RC pathology.^15^^,^^22^ A stable and asymptomatic os acromiale may become unstable and symptomatic following trauma or surgical interventions such as subacromial decompression.^13^ Therefore, preoperative identification and assessment of os acromiale are critical for optimizing surgical planning and improving postoperative outcomes. Instability of the unfused acromion fragments may increase mechanical stress on the RC, potentially resulting in RC injuries and subacromial impingement. Advanced imaging techniques, such as MRI and CT, are essential for accurate diagnosis and the development of effective treatment strategies. Although most os acromiale cases remain asymptomatic and require no intervention, MRI or CT can help identify cases with potential instability, fragment mobility, or associated subacromial bursitis. Such information may support risk stratification and guide surgical planning, particularly for procedures such as RC repair or subacromial decompression.^13^^,^^14^^,^^16^ The os acromiale fragments observed in this Japanese cohort were smaller than those reported in Western populations^5^; however, they were significantly associated with RC injuries. This finding highlights the biomechanical relevance of even relatively small fragments in individuals with proportionally smaller skeletal structures. Thus, the recognition of ethnic and anatomical variations is essential for accurate preoperative risk assessment and the development of appropriate surgical strategies in patients with os acromiale.

This study has some limitations. First, selection bias may have been introduced because only symptomatic patients who underwent MRI were included, potentially overestimating the association between os acromiale and RC injuries. Second, variability in MRI protocols across institutions and retrospective data collection may have caused information bias or inconsistent detection of os acromiale. Third, the absence of subjects with asymptomatic shoulders limited the ability to generalize findings to the broader population. Finally, the exclusive inclusion of symptomatic individuals requiring MRI evaluations introduced selection bias.

## Conclusion

Os acromiale was significantly associated with diseases involving RC injuries. This multicenter study provides new insights into the prevalence (1.1%) and clinical significance of os acromiale in the Japanese population. The prevalence in this cohort was lower than that reported in non-Asian populations, and the os acromiale fragments tended to be smaller in size compared to those observed in Europeans.

## Acknowledgment

The authors wound like to thank Editage (www.editage.com) for English language editing.

## Disclaimers:

Funding: This study was supported by 10.13039/501100001691JSPS KAKENHI Grant Number JP20K18032 and the Ogata Memorial Foundation for the Promotion of Science (2025 grant).

Conflicts of interest: The authors, their immediate families, and any research foundation with which they are affiliated have not received any financial payments or other benefits from any commercial entity related to the subject of this article.
